# Recent Progress in Torovirus Molecular Biology

**DOI:** 10.3390/v13030435

**Published:** 2021-03-08

**Authors:** Makoto Ujike, Fumihiro Taguchi

**Affiliations:** 1 Laboratory of Veterinary Infectious Diseases, Faculty of Veterinary Medicine, Nippon Veterinary and Life Science University, 1-7-1 Kyonan-cho, Musashino, Tokyo 180-8602, Japan; fumitaguchi@jcom.zaq.ne.jp; 2 Research Center for Animal Life Science, Nippon Veterinary and Life Science University, 1-7-1 Kyonan-cho, Musashino, Tokyo 180-8602, Japan

**Keywords:** torovirus, coronavirus, enteric diseases, replication, transcription, non-structural proteins, structural proteins, reverse genetics

## Abstract

Torovirus (ToV) has recently been classified into the new family Tobaniviridae, although it belonged to the Coronavirus (CoV) family historically. ToVs are associated with enteric diseases in animals and humans. In contrast to CoVs, which are recognised as pathogens of veterinary and medical importance, little attention has been paid to ToVs because their infections are usually asymptomatic or not severe; for a long time, only one equine ToV could be propagated in cultured cells. However, bovine ToVs, which predominantly cause diarrhoea in calves, have been detected worldwide, leading to economic losses. Porcine ToVs have also spread globally; although they have not caused serious economic losses, coinfections with other pathogens can exacerbate their symptoms. In addition, frequent inter- or intra-recombination among ToVs can increase pathogenesis or unpredicted host adaptation. These findings have highlighted the importance of ToVs as pathogens and the need for basic ToV research. Here, we review recent progress in the study of ToV molecular biology including reverse genetics, focusing on the similarities and differences between ToVs and CoVs.

## 1. Introduction

The genus Torovirus (ToV) belongs to the order Nidovirales, family Tobaniviridae, subfamily Torovirinae, but, historically, it belonged to the family Coronaviridae [1]. The order Nidovirales was recently subdivided into 11 families, but previously consisted of four evolutionarily related families, Coronaviridae, Arteriviridae, Roniviridae, and Mesoniviridae [1]. In this review, mentions of nidoviruses refer to these four families and to ToV.

**Figure 1:**
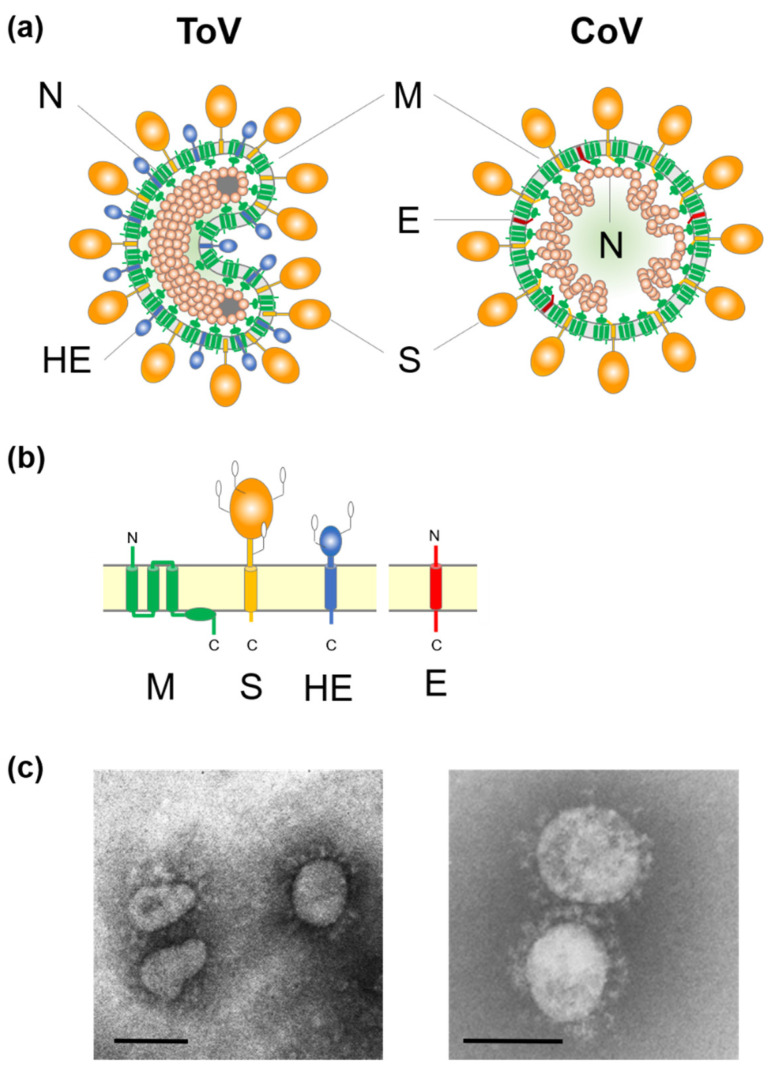
(**a**) Schematic diagrams of torovirus (ToV) and coronavirus (CoV) virions: spike (S), envelope (E), membrane (M), haemagglutinin-esterase (HE), and nucleocapsid (N) proteins. (**b**) Topology of the four structural envelope proteins. All proteins are depicted as monomers, but the S and HE proteins form homotrimers and homodimers, respectively. Oligosaccharides on the S and HE proteins are shown. Although a number are omitted, the S and HE proteins contain 19 to 28 and 7 to 12 N-glycosylation sites, respectively. (**c**) Electron micrograph of BToV (left) and SARS-CoV (right). Bar: 100 nm.

ToV is an enveloped virus with a linear, non-segmented, positive-sense single-stranded RNA genome. Torovirions exhibit polymorphisms such as spherical, oval, rod- and kidney-shaped particles [2,3,4,5] (Figure 1). Its nucleocapsid forms a helically symmetric tubular structure, a doughnut-like shape described by the Latin word ‘torus’, which is the origin of the name Torovirus [6]. ToV is thought to be predominantly associated with gastrointestinal diseases in animals and human [2,3,7,8]. Equine torovirus (EToV), the prototype of this family, was first isolated from a horse in Switzerland in 1972, bovine torovirus (BToV) was isolated from calves in Iowa in 1979, and porcine torovirus (PToV) was detected in piglets in the Netherlands in 1998 [2,3,8]. ToVs, and particularly BToV and PToV, have been detected in many countries [9,10,11]. However, the molecular biology of ToVs has been studied less intensively than that of coronaviruses (CoV), because ToV infections are generally asymptomatic or not severe and ToV is difficult to propagate in cultured cells, except for EToV. This review focuses on recent progress in ToV molecular biology, including reverse genetics, and the similarities and differences with CoVs. For clinical, epidemiological, diagnostic, and evolutionary studies of ToV, refer to the extant excellent reviews [9,10,11,12,13,14].

## 2. Animal and Human ToVs

Besides the established three ToVs (EToV, BToV, and PToV), a serological survey found antibodies against ToVs in the sera of other animals, such as goats, sheep, rabbits and mice, but the results of human serum were inconclusive [15,16]. Electron microscopy (EM) studies have found ToV-like particles in faeces from cat and human [7,17], and the full genome sequences of ToV from Tasmanian Devil [18] and goat have been reported. ToVs may infect a wide range of animal species, and the following is an overview of four representative ToVs.

### 2.1. Equine ToV (EToV)

EToV (Berne virus [BEV]) was first isolated from the rectal swab of a horse with diarrhoea in Berne, Switzerland, in 1972 (but reported in 1983) [3]. BEV was the only ToV that can be propagated in cultured cells (e.g., equine dermis [ED] cells) until BToV [19]. BEV was used for most molecular biological studies, and so is regarded as the prototype virus of this family. Repeated attempts to isolate other EToVs from horse faeces have not been successful, suggesting that BEV is a host-range mutant selected or modified to adapt to cultured cells [13]. Most horse sera in Switzerland and the sera of a few horses in Germany, France, and the United States were seropositive for BEV, suggesting that EToV are widespread worldwide [15]. Because experimental or natural infection by BEV does not induce obvious clinical symptoms, it is likely of little pathogenic importance [13,15].

### 2.2. Bovine ToV (BToV)

BToV (Breda virus [BRV]) was isolated from the faeces of calves with severe diarrhoea in Breda, Iowa, in 1979 (reported in 1982) [2]. BToV is antigenically divided into two serotypes, BRV1 and BRV2 [20]. Because cell culture of BRV was long unsuccessful [2,9], it could only be propagated in gnotobiotic or colostrum-free calves. However, human rectal tumor-18 cells, a subcell line (HRT18-Aichi), was reported to be susceptible to BToV in 2007 [19], and several other BToVs were isolated and propagated using this cell line [21,22,23]. Currently, BToV is the only virus available in the reverse genetic system [24]. Because natural and experimental infection by BToV causes diarrhoea in calves [2,20,25,26] and may be associated with respiratory symptoms [27], most clinical and pathogenic data on ToV were obtained from BToV [9,13]. BToV has been detected on all five continents [19,21,22,23,27,28,29,30,31,32,33,34,35,36,37,38,39,40,41,42,43,44,45], and a serological survey indicated that most adult cattle are seropositive [13,16,19,20,46].

### 2.3. Porcine ToV (PToV)

PToV was detected and characterised in faeces of piglets in the Netherlands in 1998 [8] but has not been propagated in cell culture. Although PToV is detected at a higher rate in piglets with diarrhoea than in healthy piglets, its clinical relevance remains unclear. Coinfections with other pathogens may exacerbate their symptoms [11]. PToV has been detected in many countries [34,47,48,49,50,51,52,53], and a serological survey showed that almost all pigs over the age of 11 weeks in Spain were seropositive [54].

### 2.4. Human ToV (HToV)

HToV was detected by EM in the faeces of adult and child patients with gastroenteritis in France and the United Kingdom in 1984 [7]. Subsequently, it has been detected in the faeces of patients with or without diarrhoea in several countries by EM, immuno-EM and enzyme-linked immunosorbent assay (ELISA) [55,56,57,58,59,60,61,62,63]. HToV shows antigenic cross-reactivity to BToV or EToV, as demonstrated by immuno-EM, ELISA, Hemagglutination inhibition test, and Western blotting [7,55,56,58]. Due to its high prevalence, HToVs are thought to be the causative agent of human diarrhoea [59,60,63]. However, cell isolation of HToV has not yet been reported. Moreover, although the full-length genome of the above three ToVs and of ToVs from Tasmanian Devils [18] and goat have been analysed, few HToV sequences are available; the 3’- untranslated region (UTR) (219 bp), which is 99% identical to that of BEV [58]; one HE gene; and several M genes are available in the National Center for Biotechnology Information database. Therefore, no standard genetic method (e.g., reverse transcriptase-polymerase chain reaction [RT–PCR]) has been established for diagnosis, and the existence of HToV requires conclusive evidence from full genome analysis or other experimental studies.

## 3. Electron Microscopy Study of ToV Morphogenesis and Double-Membrane Vesicles

### 3.1. Virion Structure and Morphogenesis

Extracellular ToV virions show polymorphisms such as spherical, oval, rod, and kidney shapes, with a diameter of 120 to 140 nm, by EM [2,3,20,25] (Figure 1c). However, native virions are likely to be bacilliform and 100–140 nm in length and 35–42 nm in width [64]. ToVs are enveloped and have two surface projections; presumably homotrimers of the CoV-like spike (S) protein (15–20 nm) and homodimers of haemagglutinin-esterase (HE) protein (5–7 nm) that may be lost upon adaptation to cell culture (Figure 1a,b). The triple membrane spanning membrane (M) protein, which resembles CoV M protein topologically, is embedded in the virion envelope. The nucleocapsid (N) proteins encapsidate the viral genome to form the helically symmetrical tubular nucleocapsid, and the characteristic toroidal structure. Studies of ToV morphogenesis were performed using BEV in ED cells and BRV in intestinal tissue from infected calves by EM [4,5]. Although extracellular ToV virions showed polymorphisms, intracellular ToV virions were of a straight, rod-like shape, and so morphological changes may occur during budding or sample preservation. ToVs bud predominantly at the Golgi apparatus and the endoplasmic reticulum (ER), thus gaining an envelope [4,5,65]. Unlike CoV, EM showed that the BEV and BRV nucleocapsids were present in the nucleus and cytoplasm of infected cells [4,5]. Subsequently, N protein accumulation in the nuclear compartments was confirmed by indirect immunofluorescence [66,67], and the importance of nuclear N accumulation for virus replication has been reported [67].

### 3.2. Double-Membrane Vesicles (DMVs)

All positive-stranded RNA viruses replicate in the cytoplasm of infected cells and modify the cellular membranes as viral replication organelles (ROs) with specialised structures [68,69]. Viral ROs are thought to play two roles; one is to concentrate viral replication proteins and relevant host factors to serve as a scaffold for viral RNA (vRNA) synthesis, and the other is to conceal double-strand (ds) RNA, the intermediate of vRNA replication, from host immune sensors [70]. All genera of CoVs (alpha, beta, gamma, and delta) [71,72,73,74,75,76] and ToV (BEV) [65] induce double-membrane vesicles (DMVs), which are derived from the ER as ROs in infected cells. In addition to DMVs, alpha- and beta-CoVs generate a complicated branching network of membranes, known as convoluted membranes (CMs) [73,74,75,76], whereas gamma- and delta-CoVs generate a nonbranching form, known as a zippered ER, and double-membrane spherules (DMSs) [71,72]. However, recent EM studies have shown that diverse CoVs (alpha-, beta-, and gamma-CoVs) induce CMs, DMSs, and DMVs, and that DMVs are the primary site of CoV vRNA synthesis in these membrane structures, suggesting that CoV genera produce essentially the same membrane structures [77]. In contrast, these additional membranous structures have not been observed in ToV-infected cells [65]. The role of these additional membranous structures of CoV in replication and why ToVs do not produce them is unclear. In addition, because infected cells usually contain completely sealed DMVs with no openings to allow the export of newly synthesised RNA to the cytosol, the mechanism of RNA transport to the cytosol is not known. Recently, a molecular pore complex that spans the double membrane and would allow the export of RNA to the cytosol was identified [78]. ToVs may form similar molecular pores. Further studies may shed light on the roles of membrane structures in CoV and ToV replication.

## 4. Genome

### 4.1. Genome Organisation 

**Figure 2:**
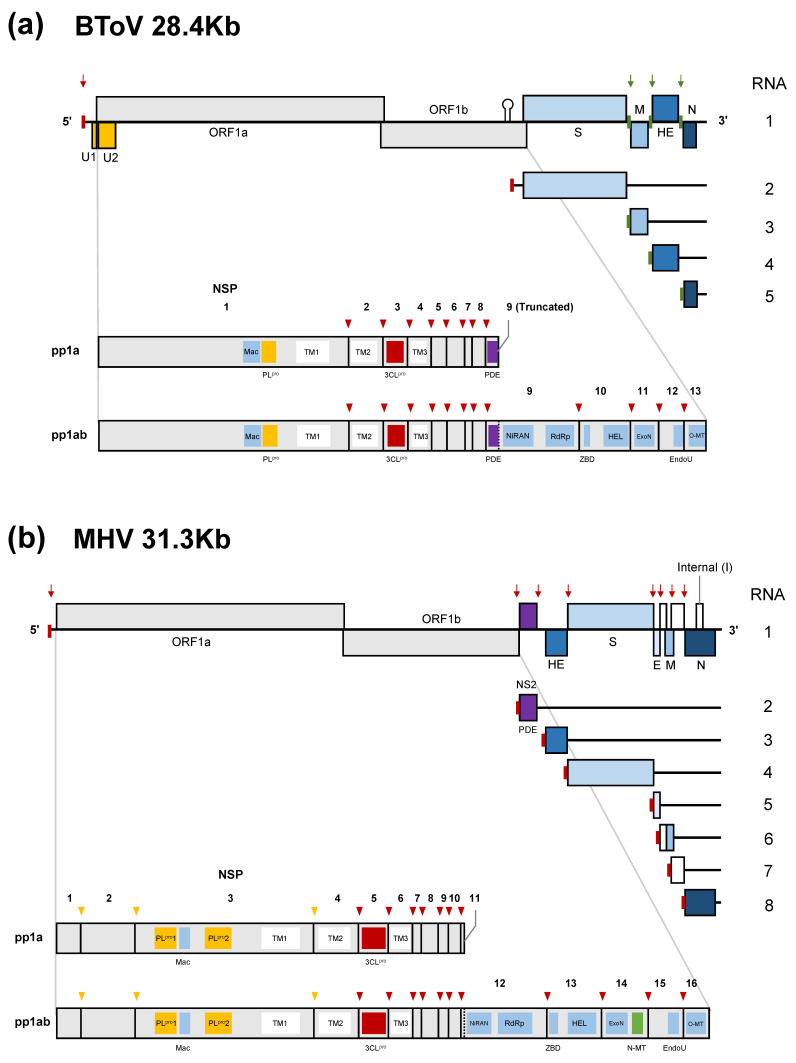
Genome organisation and gene expression of (**a**) BToV and (**b**) MHV (lineage A beta-CoV). The subgenomic (sg) mRNA species are numbered from large to small, with the genome designated RNA1. BToV and MHV polyprotein processing of pp1a and pp1ab and the conserved domains of nonstructural proteins (nsps) are shown. U1 and U2 proteins may be translated from an unconventional CUG initiation codon. The internal (I) protein is encoded within the N gene in some beta-CoVs. The 5′-leader sequence present in the genome and sg mRNA 2 of ToV and the 5′-leader sequence of CoV in all RNA species are indicated by a small red box, and terminator/promoter signals of ToV in sg mRNA3 to 5 by a small green box. Red and green arrows indicate the locations of transcription-regulating sequences (TRSs). Arrowheads indicate (deduced) cleavage sites by papain-like (orange) or 3C-like protease/main protease (red). Vertical dashed line shows a ribosomal frameshifting site. 3CL^pro^, 3C-like protease (main protease); EndoU, endoribonuclease; ExoN, exoribonuclease; HEL, helicase; Mac, macrodomains including ADP-ribose-1″phosphatase; NiRAN, nidovirus RdRp-associated nucleotidyltransferase; N-MT, guanosine N7-methyltransferase; O-MT, ribose 2′-O-methyltransferase; PDE, 2’, 5’-phosphodiesterase; PL^pro^, papain-like protease; RdRp, RNA-dependent RNA polymerase; TM, transmembrane domain; ZBD, zinc-binding domain.

ToVs have the largest, positive-sense, single-stranded RNA genome of about 28 kb with a 5’-cap structure and a 3’-poly (A) tail, which consist primarily of six conserved open reading frames (ORFs) (Figure 2a) [79,80,81]. The genome has 5’-UTR (~820–850 kb) and 3’-UTR (~200 kb), respectively. The 5’-proximal two-thirds of the genome contains ORF1a and ORF1b, encoding replicase-transcriptase proteins. These are synthesised as two large polyproteins; pp1a is translated from ORF1a and pp1ab from ORF1a/1b by -1 ribosomal frameshifting [79]. These polyproteins are proteolytically processed into 13 non-structural proteins (nsps) [82]. The remaining one-third of the genome encodes four structural (or accessory) proteins: S, M, HE, and N proteins in that order. Because HE protein is not essential for viral growth in cultured cells [19,21,22,24], it is also regarded as an accessory protein that may promote host adaptation. ToV and CoV nsps share six domains—3C-like protease (3CL^pro^), nidovirus RdRp-associated nucleotidyltransferase (NiRAN), RNA-dependent RNA polymerase (RdRp), zinc-binding domain (ZBD), helicase (HEL), and endoribonuclease (EndoU)—which are conserved in all or most nidoviruses [83,84]; exoribonuclease (ExoN) and ribose 2′-O-methyltransferase (O-MT) domains, which are conserved in all nidoviruses except arteriviruses [85,86]; and macrodomains (Mac) (previously called X domain) and papain-like protease (PL^pro^) domains. The differences between ToVs and CoVs and some remarkable characteristics of the former are as follows:

- The 3’ proximal one-third of the CoV genome encodes four structural proteins in the order 5’-(HE)-S-E-M-N-3’ (if HE is present) and contains a variety of species-specific accessory proteins. In contrast, that of ToVs encodes 5’-S-M-(HE)-N-3’ in that order, with HE as the only accessory protein.
- ToV lacks envelope (E) protein, which is important for virion assembly in CoV [87], and the N protein (~160 amino acid [aa]) of ToV is significantly smaller than that of CoV (~450 aa).
- Two deduced CUG-initiated ORFs encoding U1 and U2 proteins are found in the 5’-UTR and within ORF1a [81].
- ToVs lack guanosine N7-methyltransferase (N-MT), which is conserved in CoV and ronivirus of the nidoviruses [88].
- The 3’-end of ORF1a of ToV encodes 2’, 5’-phosphodiesterase (PDE). Interestingly, related PDE (NS2) was identified only in a lineage A beta-CoV and encoded at a different genome location, being translated from subgenomic (sg) mRNA 2 as an accessory protein (Figure 2b) [85]. Only lineage A beta-CoV have an HE gene.

### 4.2. Recombination

**Figure 3:**
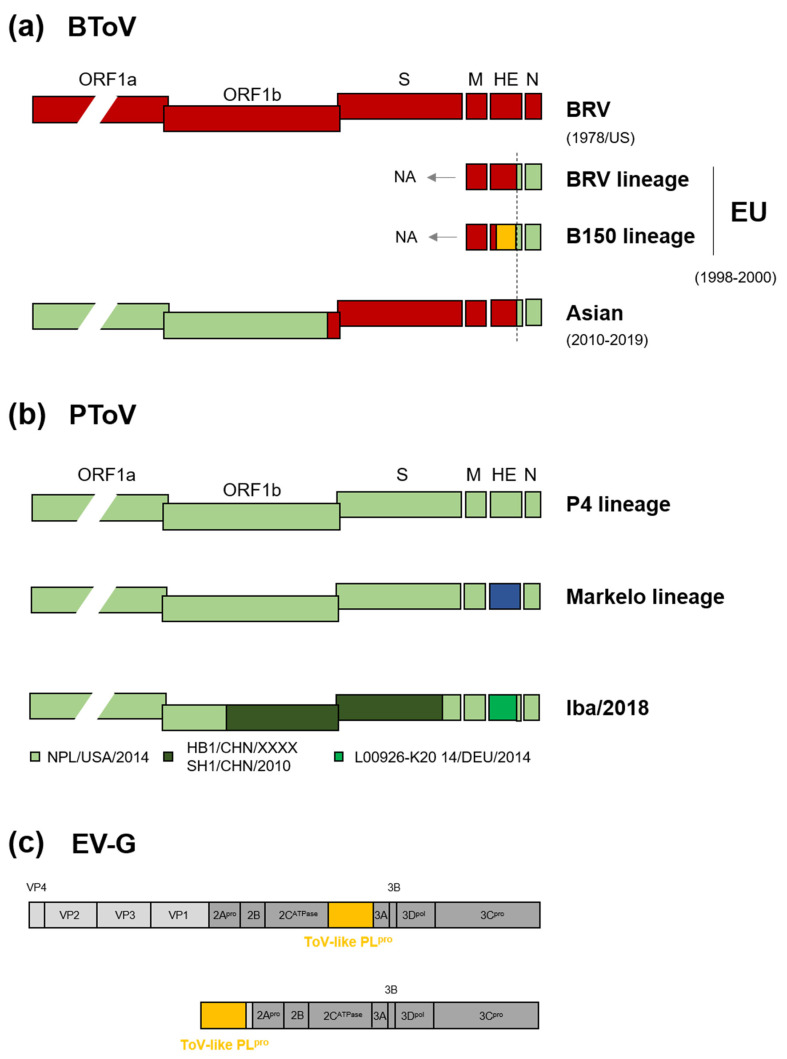
Homologous and heterologous recombination of ToVs. (**a**) Genome organisation of BToV by interspecies homologous recombination between BRV-like BToV (red) and PToV (green). Recombination of HE from an as-yet-unknown ToV is shown in orange. European and Asian isolates were from Italy, Hungary, the Netherlands, and Japan and China, respectively. Years of isolation are shown. BToV HE gene can be divided into two lineages, the BRV and B150 lineages. NA, not analysed. (**b**) Two lineages of PToV based on the HE gene and genome organisation of Japanese PToV (Iba/2018) with a mosaic sequence. The HE gene of the Markelo lineage is shown in blue. PToV (Iba/2018) may be a result of intraspecies homologous recombination among three related PToVs (named below). (**c**) Heterologous recombination between ToV and picornavirus. The ToV-like PL^pro^ gene is inserted into the EV-G genome at the 2C/3A junction or is completely replaced by a viral structural gene up to the VP1/2A junction.

There is much evidence for heterologous and homologous recombination of ToVs. A full-length genome analysis of recent Japanese BToV indicated interspecies recombination between BRV-like BToV and PToV at the 3’-ends of ORF1b and the HE gene (Figure 3a) [23,42]. An identical recombinant breakpoint in HE was found in European BToVs isolated in the late 1990s [89], suggesting that European and Japanese BToVs share a common ancestor. Due to the lack of sequence information on the 5’ proximal end of European BToVs, it is unclear whether recombination at the 3’-ends of ORF1b had already or later happened. Moreover, additional HE recombination was observed in European BToVs (Figure 3a) [89]. The HE gene of BToV can be genetically divided into two lineages, the BRV and B150 lineages [53,89]. A full-length genome analysis of five PToVs showed that they can be divided into two lineages, the Malkelo and P4 lineages, based on the HE gene [80,90,91], but not other genes [53] (Figure 3b). The two lineages may have been separated by an HE recombination event [89], and their common ancestor reportedly emerged 56 years ago [80]. Recent Japanese PToVs have a mosaic sequence comprising three related PToVs, presumably a result of intraspecies recombination (Iba/2018 in Figure 3b) [53]. Due to the frequent recombination of the HE gene of BToV and PToVs [53,80,89], this region appears to be a recombination hotspot.

Heterologous recombination might lead to relocation of the HE and PDE genes in ToV and a lineage A beta-CoV. Their HE, which is related to haemagglutinin-esterase-fusion (HEF) protein of influenza C, and PDE are thought to have been independently obtained from these ancestors by heterologous recombination events (Figure 2) [92]. Remarkably, heterologous recombination between ToV and picornavirus has been discovered recently in many countries; a ToV-like PL^pro^ gene is inserted into the porcine enterovirus G (EV-G) genome at the 2C/3A junction [93,94,95,96,97] or completely replaces a viral structural gene up to the VP1/2A junction [98,99] (Figure 3c), suggesting that ToV-like PL^pro^ benefits the growth of EV-G.

### 4.3. Replication and Transcription

**Figure 4:**
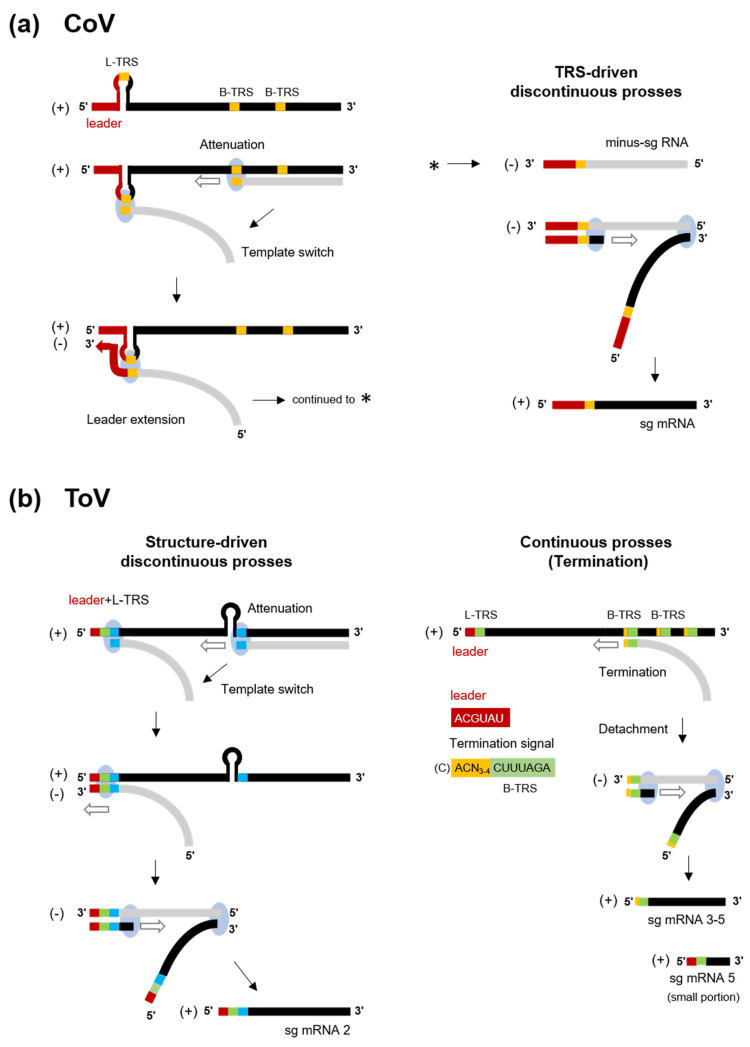
Discontinuous and continuous transcription of CoV and ToV. (**a**) Model of TRS-driven discontinuous transcription of CoV. (-) RNA synthesis (light grey) initiates at the 3’-end of the genome and is terminated or attenuated when the replication and transcription complex (light blue circle) encounters B-TRS (in orange). The discontinuous step is driven by base-pairing between the anti-B-TRS and L-TRS within the hairpin, resulting in template switch from nascent (-) RNA to the leader. Next, (-) RNA synthesis re-initiates and a leader sequence (red) is added. This complete (-) sg RNA in turn serves as a template for (+) sg mRNAs. (**b**) Model of structure-driven discontinuous (left) and continuous (right) transcription of ToV. In sg mRNA2, nascent (-) RNA synthesis is terminated by the hairpin (HP) structure and base pairing between homologous regions (blue), followed by L-TRS (green) and HP, promotes the template switch. After switching, the 5’ genome-derived sequence including a short leader (red) and L-TRS and additional nucleotides are added to nascent (-) RNA, resulting in complete (-) sg RNA synthesis. In sg mRNA 3-5, semiconserved sequences in the genome, CACN_3-4_CUUUAGA (yellow and green) including B-TRS (green) act as a termination signal, and complete (-) sg RNA synthesis is terminated at this region and is then detached from genome. (+) sg mRNA contains ACN_3-4_CUUUAGA at the 5’-end (without the C residue of the 5’-genome). A small portion of mRNA 5 contains a leader sequence that is subjected to discontinuous transcription.

In nidoviruses, RNA-dependent RNA synthesis mediates genome replication and transcription of a nested set of 3’-coterminal sg mRNAs. Genome replication is initiated by the synthesis of full-length minus-sense (-) genomic copies, from which new positive-sense (+) genomic RNA is generated. The replicase polyproteins pp1a/pp1ab are expressed from genomic RNA, and the structural or accessory proteins are expressed from a set of sg mRNAs (Figure 2). With a few exceptions, sg mRNAs are structurally polycistronic but functionally monocistronic, and only the 5’-proximal ORF is translated. In CoVs (and arteriviruses and the basically similar mesoniviruses [100]), all sg mRNAs have a common leader sequence (65–98 nt) at their 5’ end [101,102,103,104] (Figure 2b). Therefore, CoV sg mRNAs are synthesised by fusion of the leader and the 5’-end of each mRNA-coding sequence (called the “body”) by a characteristic, discontinuous transcription process [101,104]. This process is controlled by the transcription-regulating sequence (TRS) adjacent to the leader sequence (leader [L]-TRS), and TRSs located upstream of each ORF in the 3′ one-third (body[B]-TRSs) [101,104] (Figure 2b and Figure 4a). The L-TRS is located within a loop of a conserved hairpin structure that is associated with replication and transcription [105,106,107]. During (-) RNA synthesis, because B-TRSs act as signals for attenuation or termination of nascent (-)RNA synthesis, after the replication and transcription complex (RTC) encounters B-TRSs, the synthesis of nascent (-) RNA stops and is reinitiated at the L-TRS. This discontinuous step is driven by base-pairing between the L-TRS and anti B-TRS [108], resulting in a template switch from nascent (-) RNA to the leader. Next, a (-) leader sequence is added to the nascent (-) RNA, leading to complete (-) sg RNA synthesis. Each (+) sg mRNA is generated from the corresponding (-) sg RNA template [101,104]. 

Two sg mRNAs of ronivirus do not have a common leader sequence at the 5’-end, indicating continuous transcription [109]. There are conserved B-TRSs but no equivalent L-TRS in the genome, implying that its B-TRSs act as termination signals but do not promote template switch and reinitiation. 

In contrast, the transcription strategy of ToVs is unique among the nidoviruses, which employ a combined discontinuous and continuous transcription process to produce a set of sg mRNAs [110]. The genome of EToV (BEV) contains L-TRS and B-TRSs, which are highly conserved (CUUUAGA). B-TRSs are located upstream of each ORF, but there is no equivalent B-TRS of S ORF (Figure 2a and Figure 4b). Despite the possibility of base pairing between L-TRS and anti-B-TRS, mRNAs 3, 4, and 5 lack a common leader sequence and have a consensus ACN_3-4_CUUUAGA sequence (the extended TRS of mRNA) at the 5’-end identical to the viral genome. This semiconserved sequence (C)ACN_3-4_CUUUAGA (the extended TRS on genome with additional C residue at a 5’ terminal), including B-TRS and preceding each N, M, and HE ORF, acts as a termination signal of the nascent (-) RNA, and is not involved in template switch (Figure 4b right) [110,111]. Therefore, these sg mRNAs are synthesised by continuous transcription. Despite the lack of base pairing between TRSs, mRNA 2 is synthesised by discontinuous transcription. The EToV genome also contains a conserved hairpin (HP) structure in ORF1b, and a 23 nt homologous sequence following L-TRS and HP. In sg mRNA2, HP is thought to attenuate or terminate nascent (-) RNA, and base pairing between homologous regions promotes the template switch (Figure 4b left). After switching, a total of 14–18 nucleotide (nt) of 5’ genome-derived (-) sequences comprising a short leader (6 nt, ACGUAU), L-TRS (CUUUAGA), and an additional sequence (AGUUU: underlined bases cannot not be identified from which template are used) are added to nascent (-) RNA [110]. Interestingly, it has been reported that a small proportion of sg mRNA5 is synthesised discontinuously [81]. Even in this case, base pairing between TRSs is not used for the template switch, with which a short AGAA sequence adjacent to or within TRSs may be associated. Therefore, various transcription mechanisms are employed by the nidoviruses and that of ToV is particularly complex. Reverse genetics of ToV (described later) provide a good starting point for studying the functions of transcriptional elements, including HP, L-TRS, B-TRS and homologous regions.

## 5. Non-Structural Proteins (nsps)

In CoV, 15 to 16 nsps are proteolytically produced from pp1a/pp1ab by viral proteases, and most (nsp2 to 16) assemble into a membrane-associated RTC containing N protein and numerous host proteins [112,113,114,115,116]. These nsps possess multiple domains conserved or semiconserved in nidoviruses, and have enzymatic and functional activities, such as proteases, deubiquitination, polymerase, helicase, exo- and endo-nuclease, N7- and 2′O-methyltransferases, and DMV formation [69,85,112,114,115]. The roles of these domains have been elucidated in detail using several CoVs, and reverse genetic approaches demonstrate their importance for viral replication [117,118,119,120,121]. In ToVs, 13 nsps are produced in a similar manner to those of CoVs. nsp9 may comprise two forms, a truncated form including only PDE (generated from pp1a), and a full-length form including PDE and RdRp (produced from pp1ab) (Figure 2a). In contrast to CoVs, although the functions of ToV nsps are unclear, the functions of domains shared with CoVs are thought to be similar or identical. Here, we focus on known ToV nsps and those postulated to suppress the innate immune responses.

### 5.1. 3C-like Protease/Main Protease (3CL^pro^/M^pro^)

ORF1a of nidoviruses encodes 3CL^pro^ and PL^pro^, both or the former of which are/is involved in the processing of replicase polyproteins, resulting in generation of functional nsps (Figure 2). 3CL^pro^ (also termed main protease [M^pro^] because it mediates most processing) has a cellular chymotrypsin-like fold and is related to picornavirus 3C protease. Because 3CL^pro^ of nidovirus is located at a similar constellation in pp1a, flanked by two transmembrane domains (TMs), and is essential for viral replication, one might think they are genetically well conserved and evolutionally related. However, the overall sequence homology is low and their similarity is limited around the catalytic residues [85,122,123,124,125,126]. Even in catalytic sites, arterivirus 3CL^pro^ is a serine protease with a canonical Ser-His-Asp triad, which is similar to that of chymotrypsin, and recognises Glu at the P1 position (P1-Glu) as a cleavage site [127,128]. In contrast, 3CL^pro^ of CoV and ronivirus are cysteine proteases with a Cys-His catalytic dyad that recognise Gln (P1-Gln) and P1-Glu, respectively [125,126,129,130]. Remarkably, although 3CL^pros^ of ToV and CoV are the most evolutionally related, ToV 3CL^pro^ is a serine protease, like arterivirus, but employs a Ser-His catalytic dyad that recognises P1-Gln, like CoVs [82,123]. Due to the close evolutionary relationship between ToV and CoV, a transition from Cys-His of CoV to the Ser-His of ToV (or vice versa) is thought to have occurred rather than from Ser-His-Asp of arterivirus. Actually, ToV 3CL^pro^, in which Ser is replaced by Cys retains partial enzymatic activity [123].

The P1, P2, and P1’ positions in CoV 3CL^pro^ are the main determinants of substrate specificity [122,131,132] and the consensus can be expressed as LQ↓(S, A) (arrow, cleavage site); P1-Glu is highly conserved, P2 tolerates hydrophobic amino acids with a preference for Leu, and P1’ tolerates small residues such as Ser or Ala. The consensus of ToV 3CL^pro^ can be represented as FxxQ↓(S, A); P1 and P1’ are similar to those of CoVs, and Phe of P4 is a key determinant but Tyr/Met/Leu/Ile may be acceptable [82,85,123]. Although both viruses preferentially recognise P1-Glu, ToV 3CL^pro^ S1 in the substrate-binding pocket, the most important position for P1-Glu recognition, resemble those of arteriviruses and roniviruses rather than CoVs.

Interestingly, ToV 3CL^pro^ has different self-processing substrate specificities at the N- and C-terminal sites. Because most nidoviral 3CL^pros^ require P1-Glu or P1-Gln for substrate specificity, P1-Ala substitution inhibits the C-terminal self-processing of ToV 3CL^pro^. However, P1-Ala substitution only slightly affected the N-terminal self-processing and both P1-Ala and P4-Ala substitutions impair it [82]. Differences in N- and C-terminal self-processing substrate specificities have also been observed in severe acute respiratory syndrome coronavirus (SARS-CoV), albeit with different mechanisms [131]. Therefore, the evolutionary history of nidoviral 3CL^pro^ is complicated, and ToV 3CL^pro^ appears to have evolved independently and has different properties than other nidovirus 3CL^pros^.

### 5.2. Papain-Like Protease (PL^pro^)

CoVs, ToVs, and arteriviruses encode one or more PL^pro^ cysteine proteases as an accessory protease at the N-terminus of pp1a, which have little sequence similarity and are of very different sizes. Alpha- and most beta-CoVs have two PL^pro^ domains, whereas ToV and gamma- and delta-CoVs have a single PL^pro^ [85,133]. CoV PL^pro^ is critical for processing the N-terminal end of pp1a by the cleavage sites (LxGG↓), but it is unclear whether ToV PL^pro^ is involved in pp1a processing. In addition to proteolytic activity, CoV PL^pro^ has deubiquitination (DUB) and deISGylation activities. Because ubiquitination is involved in regulation of innate immune signalling pathways, the DUB activity of PL^pro^ cleaves the C-terminal LRGG↓motif of ubiquitin, mediating its removal from target proteins, which may disturb immune signalling and block induction of the antiviral state. By contrast, ISG15 is an interferon (INF)-stimulated ubiquitin-like molecule that can be linked to cellular proteins by ISGylation [134], which is thought to be important for induction of an innate immune response. CoV PL^pro^ can also remove IGS15 conjugates from cellular proteins (deISGylation). Therefore, the DUB and deISGylating activities of CoV PL^pro^ may play a role in antagonism of the innate immune response to viral replication (reviewed in [133]). Although ToV PL^pro^ has not yet been studied, exogenous ToV-like PL^pro^, which EG-V acquired by heterologous recombination and shows 54 to 68% amino acid sequence identity to a canonical ToV PL^pro^, has DUB and deISGylation activities and suppresses IFN-β induction [95]. Therefore, ToV PL^pro^ likely suppresses the innate immune response to promote viral replication.

### 5.3. Capping Enzyme

5’ Capping of viral RNAs is important not only for translation of viral proteins but also for escape from the innate immune system, which can detect non-self RNA, such as uncapped RNA and dsRNA [135]. CoV capping involves the sequential activities of four enzymes in the conventional capping pathway [135]: (i) RNA triphosphatase (RTPase) in the nsp13 helicase hydrolyses the γ-phosphate of the nascent mRNA (pppN-RNA; N represents the first nucleotide of the 5’-end and p represents a phosphate group) [136]; (ii) an as-yet-unidentified guanylyltransferase (GTase) transfers a GMP molecule (Gp) to the 5’-diphosphate RNA (ppN) to create GpppN; (iii) N7-MTase (nsp14) methylates the cap guanosine to form the basic cap-0 structure (m7GpppN), which is the minimum structure recognised by the translation factor eIF4E [137]; and (iv) 2O-MTase (nsp16) carries out additional methylation of the first or second nucleotide, generating the cap-1 (m7GpppNm2) or cap-2 structure [138], which are required to evade the non-self RNA recognition system of the innate immune response [139,140]. Although 5’ capping and methylation have been experimentally characterised using CoVs, a bioinformatics analysis indicated that all nidoviruses except arteriviruses have a 2O-MTase in their pp1ab and that all large-genome nidoviruses except ToV encode an N7-MTase [85]. However, attempts to detect N7-MTase activity in the corresponding proteins of roniviruses and mesoniviruses and to identify proteins with N7-MTase activity in ToV have failed [86]. A protein containing 2O-MTase activity was experimentally confirmed in ronivirus, while that of ToV did not show 2O-MTase activity [86]. This failure may be due to the detection limit of the assay or a requirement for other proteins, because nsp10 acts as a cofactor for 2O-MTase activity in CoV. Therefore, the proteins involved in 5’-cap methylation of ToV have not yet been experimentally identified and confirmed.

### 5.4. 2′,5′- Phosphodiesterase (PDE)

The C-terminus of pp1a in ToV and accessory protein NS2 of lineage A beta-CoV are homologous proteins with PDE activity, although they are encoded at different locations in the genome (Figure 2) [85]. When dsRNA, the viral replication intermediate, in infected cells is detected by the innate immune system and an INF response is induced, 2’, 5’-oligoadenylate synthetase (OAS) generates 2’, 5’-oligoadenylate (2-5A), which activates RNase L to degrade host and viral single-strand RNA and terminate protein synthesis, subsequently inducing apoptosis [141]. Because CoV NS2 protein cleaves 2-5A, it counteracts host IFN signalling by antagonising the OAS/RNaseL pathway [142]. NS2 is a critical determinant of murine hepatitis virus (MHV) liver tropism in mice, indicating involvement in viral pathogenesis [142]. The corresponding protein of ToV has PDE activity, preventing activation of RNase L, and can complement an inactive MHV NS2 gene [143]. ToV and lineage A beta-CoV share a common strategy of antagonising the OAS/RNase L antiviral pathway by means of their PDEs, and the ToV PDE in nsp9 may be involved in pathogenicity.

### 5.5. U1 and U2 Proteins

A comparative genome analysis and ribosome profiling of ToV reveal two conserved novel ORFs, from which unknown function U1 and U2 proteins might be translated from an unconventional CUG initiation codon in the 5’-UTR and in another frame of ORF1a, respectively (Figure 2a), although the expression of these proteins has not been experimentally confirmed [81,144]. The highly basic U1 (~10 kDa) and U2 (~30 kDa) have no homology to any other proteins in public databases. Because U1 protein contains two predicted TM domains connected by a short hinge, one potential function from structural similarity to known protein is the viral ion channel. Because ToV lacks E protein (~10 kDa) with a single TM domain, which has ion channel activity and is involved in virion assembly in CoV, U1 protein may have a similar function as CoV E protein. In contrast, the function of U2 could not be predicted. Analysing whether these two proteins are expressed in infected cells and, if so, what are their functions, is an interesting topic for future research. In addition, the difference in the locations of the overlapping genes between CoV and ToV is interesting. The overlapping CoV genes are located in the 3’-proximal one-third of the genome encoding structural or accessory proteins; for example, the internal (I) protein encoded within the N gene of some beta-CoVs is translated from AUG on sg mRNA by leaky scanning [145,146,147]. By contrast, the overlapping ToV gene U2 is within ORF1a and is presumably translated from an unconventional CUG in the genome. Analysing the localisation and presence of overlapping genes may provide clues to the evolution of ToV and CoV.

## 6. Structural Proteins

ToV and CoV have three common structural proteins, the S, M, and N proteins (Figure 1a,b). Although they have different primary sequences, the S and M proteins show topological and structural similarities, being evolutionally related, whereas N protein, which is of a significantly different size, is probably unrelated. ToV and some beta-CoVs share HE protein, which is incorporated into virions as a structural protein [148] and is also regarded as an accessory protein because it is not essential for viral growth in cell culture [19,21,22].

### 6.1. Spike (S) Protein

**Figure 5:**
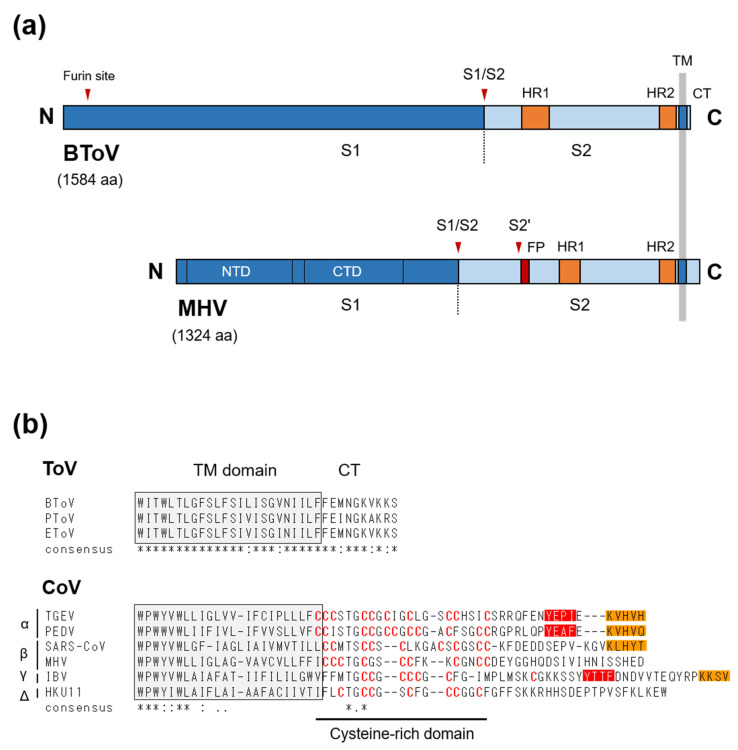
(**a**) Schematic diagrams of the S proteins of ToV and CoV. BToV contains two furin sites, an S1/S2 site and an additional site near the N-terminal end, whereas MHV (CoV) has two well-defined protease cleavage sites, S1/S2 and S2′ (arrowheads). The S protein consists of two subunits, the S1 receptor-binding subunit and the S2 fusion subunit. NTD: N-terminal domain of S1, CTD: C-terminal domain of S1, S1/S2, and S2′ cleavage sites, furin-site: Additional furin cleavage site, FP: putative fusion peptide, HR1: heptad repeat 1, HR2: heptad repeat 2, TM: transmembrane domain, CT: cytoplasmic tail. (**b**) C-terminal ends of three ToV and six CoV S proteins. Amino acid sequence alignment was performed using the ClustalW program. Shaded box indicates the deduced TM domain. Cysteine residues in the cysteine-rich domain of CoVs are indicated in red. Orange and red boxes indicate potential ER retrieval signals (KxHxx- or KKxx-motif) and tyrosine-dependent localisation signals/internalisation signals (Yxxθ motif, where θ can be F, I, L, M, or V), respectively.

The S protein (originally known as the peplomer [P] protein) of ToV is a type I glycoprotein of about 180–200 kDa (~1580 aa), with 19 to 28 potential N-linked glycosylation sites [149,150]. Although a sucrose gradient assay showed that S protein formed homodimers in infected cells, it may form homo-trimers because of its similarity to other type-I viral fusion proteins [149]. S protein is responsible for cell attachment and fusion of the viral and cellular membranes during entry, and so is a crucial determinant of tissue and cell tropism as well as host range [151,152,153,154]. The aa sequence of ToV S protein shows structural characteristics of typical type-I viral fusion proteins, including CoV S protein: An N-terminal signal sequence, C-terminal TM, a short cytoplasmic tail (CT) as well as two heptad repeats important for fusion activity, and a furin-cleavage site (S1/S2) by which the precursor S0 is processed into two S1 and S2 subunits (Figure 5a). ToV S1 subunit would have a receptor-binding domain, and S2 subunit must have an as-yet-undefined fusion peptide essential for fusion activity. Due to the essential roles of S protein in viral infection, although receptor identification, cell entry, and three-dimensional structure studies of CoVs have been performed, little attention has been paid to those of ToVs. With regard to cell attachment, the functional receptor of ToV is unknown, and information on cell attachment factors is sparse. Cell-adapted EToVs or BToVs that lost their HE proteins showed haemagglutination activity via S proteins, with some differences in species preference. Human, rabbit, and guinea pig, but not rat, goose, chicken, or horse red blood cells (RBCs), were haemagglutinated by cell-adapted EToV [155], whereas haemagglutination was observed by cell-adapted BToV with mouse and rat but not with chicken, turkey, goose, cow, horse, or guinea pig RBCs [19,21,156]. COS7 cells expressing the BToV S protein alone showed haemadsorption to rat RBCs, which was lost after neuraminidase (NA) treatment of the RBCs, suggesting S protein-mediated binding requires sialic acid (Sias). By contrast, pretreatment of permissive HRT-18 cells with NA did not affect the efficiency of BToV infection [156]. Although the role of Sias in cell attachment via ToV S protein needs further study, Sias are not likely to be essential for BToV entry, at least to cultured cells.

Host proteases play very important roles in the fusion activity of CoV S protein [153]. CoVs can use two distinct pathways, via fusion activity either directly at or near the cell surface or through the endosomal compartment, depending on the localisation of host proteases [157,158]. CoV S protein contains two distinct proteolytic cleavage sites, S1/S2 and S2’ located upstream of the putative fusion peptide, both of which are recognised by a variety of host proteases to activate S protein [153]. Although the proteolytic S-activation mechanisms vary among CoV species, that of SARS-CoV has been fully characterised. Without extracellular proteases, SARS-CoV was internalised through the endosomal compartment, and the S protein was activated by endosomal protease cathepsin L, leading to fusion of the endosomal and viral membranes [159]. In contrast, extracellular proteases such as trypsin, thermolysin and elastase, or the membrane-bounded transmembrane protease, serine (TMPRSS) family (predominantly expressed in the respiratory tract), could activate SARS-CoV S protein, leading to fusion of the viral and plasma membranes at or near the cell surface [157,160]. Moreover, protease-mediated entry by trypsin and thermolysin increased infection efficiency 100-fold over endosomal cathepsin-mediated entry, whereas protease pretreatment of SARS-CoV prior to receptor binding inactivated the S protein [157]. Cleavage sites analysis indicated that cathepsin L cleaved S1/S2 [161], and elastase cleaved S2’ [162]. Trypsin, which induces efficient cell–cell fusion (syncytium formation) and virus–cell fusion, is thought to cleave both sites sequentially—S1/S2 followed by S2’ cleavage. Although a different mechanism may be employed for cell–cell and virus–cell fusion, S2’ cleavage is indispensable for fusion activity, but S1/S2 cleavage seems not to be essential for cell–cell or virus–cell fusion. Similarly, it has been reported that Middle East respiratory syndrome coronavirus (MERS-CoV) can use two pathways based on endosomal cathepsin L and cell surface-expressed TMPRSS2 to activate S protein, and overexpression of the latter increases infectivity 100-fold compared to the control [163]. Also, furin, which preferentially recognises the R-x-R/K-R motif (RxxR as the minimal motif), cleaved both sites of MERS-CoV S protein with S1/S2 cleavage during S-synthesis and S2’ cleavage during viral entry [164], resulting in viral entry at the early endosome [165]. Overall, host proteases mediate cleavage during various infection steps, inducing cell–cell or cell–viral fusion and regulating infectivity, pathogenicity and host range. In contrast, little is known of the cell entry and proteolytic S-activation mechanisms of ToV, but there are some differences from CoVs. (i) The ToV S protein has a single cleavage site (a furin site), which is involved in fusion activity. Although here termed S1/S2, it likely functionally corresponds to the S2’ site of CoV, which is essential for fusion activity. By contrast, the second conserved furin site is present at the N-terminus of the S1 subunit in ToVs and is probably not involved in fusion activity (Figure 5a). The function of the second furin site in viral growth is unknown, but the S1/S2 furin site was processed in infected cells [149] and a BToV S mutant with alanine substitutions at this site was not cleaved when expressed alone in HEK293T cells [166]. (ii) BToV or EToV-infected cells or COS7 cells expressing BToV S protein did not induce syncytium formation upon exposure to low pH [12] or a protease such as trypsin [166]. (iii) In contrast to the inactivation of SARS-CoV by protease pretreatment, trypsin or chymotrypsin pretreatment of EToV prior to cell inoculation increased infectivity 5- to 10-fold [167], similar to FCoV [168]. EToV was not inactivated at pH 2.5 to 10 [167]. (iv) The boundary between a CT and TM of CoV S protein contains a cysteine-rich domain modified with palmitic acids, which is important for fusion activity [169], while those of ToVs lack cysteine residues (Figure 5b).

Another interesting difference between CoV and ToV is their CT length: the deduced CT of CoVs ranges from 35 aa (bovine coronavirus [BCoV]) to 48 aa (infectious bronchitis virus [IBV]), while that of ToV is only 10 aa. (Figure 5b). CoVs or ToVs assemble at and bud from the intracellular compartments, ER-Golgi intermediate compartment (ERGIC), or Golgi apparatus (or ER), and membrane proteins such as the S and M proteins must be retained near such intracellular compartments for efficient virion assembly [170]. In the cases of CoVs, the M protein is essential for virion or virus-like particle (VLP) formation. The CoV M protein, which contains an intrinsic retention signal, accumulates near the ERGIC and is cooperated with E protein, which is also important in virion assembly, to form virions. The S protein is dispensable for virion formation, but its presence enables S-incorporation into virions via the M–S interaction [171]. The CT of CoV S proteins contains several functional domains for efficient S incorporation. The cysteine-rich domain or its palmitoylation was required not only for fusion activity but also for the M–S interaction and S incorporation into virions [172,173]. Moreover, although it depends on virus species, the CT of some CoV S proteins possess an intracellular retention or targeting signal: An ER retrieval signal (KKxx- or KxHxx-COOH) and tyrosine-dependent localisation signal (YxxI or YxxF motif), which promote S accumulation near the ERGIC and its subsequent incorporation into virions [170,174,175,176,177,178]. For example, the S proteins of transmissible gastroenteritis coronaviruses (TGEV) and IBV, which have two signals, were primarily retained intracellularly when expressed independently [176,177], but that of MHV, which lacks signals, was primarily transported to the cell surface. However, when co-expressed with M protein, it was retained near the budding site [179]. In contrast, the CT of ToV does not contain any such signals or domains. In fact, BToV S protein was primarily transported to the cell surface when expressed alone in COS7 cells [166]. However, a pulse-chase experiment on EToV S protein using a vaccinia expression system showed that intracellular transport from the ER to the medial Golgi and cleavage of the S protein was slow, suggesting that EToV S protein possesses an intrinsic retention mechanism [149]. Our preliminary data showed that the CT truncation of BToV S protein affected its subcellular localisation, suggesting that the CTs of ToV S proteins regulate intracellular transport [166]. Further study on the role of ToV CT in intracellular transport, M-S interactions, and S incorporation into virions is needed.

### 6.2. Haemagglutinin-Esterase (HE) Protein

The HE protein is the most extensively studied ToV protein, and the only protein whose crystal structure has been solved [180]. ToV, CoV, and influenza C may have obtained their HE or HEF genes by heterologous RNA recombination independently, because these genes show 30% sequence identity each, but the origin of the HE gene is unknown [92]. The HE proteins of ToV and CoV are type-I glycoproteins of about 65 kDa (~400 aa), with 7 to 12 potential N-linked glycosylation sites and form a homodimer. By contrast, the related HEF protein forms a homotrimer. Both HE proteins have two reversible functional domains—one binds O-acetylated sialic acids (O-Ac-Sias) and the other destroys this binding. Because O-Ac-Sias is a functional receptor for some beta-CoVs and likely acts as a cell attachment factor for multiple CoVs and ToVs, the receptor-destroying activity of HE protein would promote release of progeny viruses from infected cells and prevents self-aggregation or attachment to non-permissive cells. Because most HE genes of CoVs and ToVs are strictly maintained in field strains, HE proteins must benefit viral replication under field conditions.

However, in cultured cells, there are significant differences in the roles of the HE proteins of CoVs and ToVs. Among lineage A beta-CoVs, the betacoronavirus-1 species, such as human coronavirus OC43 (HCoV-OC43) and BCoV, or human coronavirus HKU1 (HCoV-HKU1) species, use O-Ac-Sias as the principal receptor or attachment receptor determinant via the S protein [181,182]. These viruses therefore bind to O-Ac-Sias via both their S and HE proteins or only S protein (because HE protein of some HCoVs has lost its receptor-binding activity), and the receptor-destroying activity of HE protein plays an important role in virus growth. HCoV-OC43 HE protein was reportedly essential for the efficient release of progeny viruses from infected cells [183], and that the balance between the receptor-binding and receptor-destroying activities of HE proteins contributed to human adaptation by HCoV-OC43 and HCoV-HKU1 [184]. In contrast, murine coronavirus species, MHV, the S protein of which employs the murine carcinoembryonic antigen cell adhesion molecule as the principal receptor [185] and in which HE protein binds to O-Ac-Sias exclusively [186], does not require HE protein for growth in cultured cells [187,188]. Indeed, HE protein confers a selective disadvantage [189]. Similar observations have been reported for ToVs. As mentioned above, ToV S protein showed haemagglutination activity via Sias binding, but Sias were not essential for cell entry. In fact, cell-adapted strains of BToV and EToV usually failed to produce HE proteins [19,190], and BToV lost its HE gene by stop-codon insertion somewhere during serial cell passages [19,21,22]. Therefore, ToV HE protein is dispensable for, and may suppress, virus growth in cultured cells.

The HE proteins of ToV and CoV bind to O-Ac-Sias but have different substrate specificities. In most CoVs and ToVs, the HE ligand and substrate were 9-O-Ac-Sias (type I specificity) [92,191], while the specificity of one MHV biotype (MHV-S strain) shifted toward 4-O-Ac-Sias (type II specificity) [186,192]. Among the type I specificity group, further substrate preferences were observed—PToV HE protein preferentially targeted 9-mono-O-Ac-Sias, but BToV and BCoV HE proteins showed a preference for 7,9-di-O-Ac-Sias [191,193]. These preferences may be associated with host adaptation and/or tissue tropism, and the shared preference of BToV and BCoV for 7,9-di-O-Ac-Sias may result from convergent evolution for a bovine host [193]. 

Structural studies revealed that the BToV and PToV HE proteins comprise three domains: A receptor-binding jelly-roll lectin domain, an esterase domain with a Ser-His-Asp catalytic triad, and a small membrane-proximal domain, which is similar to the overall arrangement of BCoV HE [180]. In the receptor-binding site, the architecture and ligand-binding mode of PToV and BToV HE proteins are similar but different from those of BCoV HE protein. For example, the BCoV and ToV HE proteins bind specifically to 9-O-Ac-Sia by filling the critical 9-O-acetyl group into a hydrophobic pocket, but the residues comprising this pocket are not conserved and are formed from different segments. At the esterase active site, the amino acid composition of PToV HE protein closely resembles that of BCoV HE protein, but unique amino acid changes are present in the BToV HE protein. The different substrate specificities of BToV and PToV can be explained by a single amino acid. In fact, the substitution of PToV HE Thr at position 73 with Ala or Ser resulted in loss of substrate specificity, whereas substitution of BToV HE Ser at the corresponding position for Thr shifted the substrate preference from 7,9-di-O-Ac-Sias to 9-mono-O-Ac-Sias [180].

The transitions in ligand and substrate specificity (e.g., from 9-O-Ac-Sias to 4-O-Ac-Sias) would require coevolution of two different lectin and esterase domains in HE protein [194]. In addition, if the S protein has O-Ac-Sias binding activity as a principal receptor, S and HE proteins need to be functionally interdependent and must have coevolved to share a substrate specificity [184,195]. Therefore, it is an interesting research topic whether the haemagglutination activity of ToV S protein is associated with O-Ac-Sias binding or whether the ToV S and HE proteins are now or were previously functionally related.

### 6.3. Membrane (M) Protein

The M protein (originally known as the envelope [E] protein) of ToV is triple-spanning envelope protein of about 22 kDa (~233 aa; 26.5 kDa by mass calculation but 22 kDa by electrophoresis) without a conventional signal sequence, which is topologically similar to CoV M protein. ToV M protein is a non-glycosylated but CoV M protein is glycosylated in the N-terminal ectodomain. The M proteins of CoVs and ToVs are believed to share structural characteristics; a short N-terminal ectodomain, three TM domains, and a long C-terminal CT domain consisting of a closely membrane associated, amphipathic domain, and a short domain at the tail end [196]. As mentioned above, the CoV M protein is essential for virion assembly and has an intracellular retention signal [170]. In infected cells or single M-expressing cells, CoV M proteins accumulate predominantly at the Golgi apparatus, for which the first of the three TM domains is indispensable [197,198,199]. In ToV, M protein tagged with a peptide derived from MHV at the C-terminus using a vaccinia expression system was retained intracellularly but predominantly at the ER rather than the Golgi apparatus as for CoV, suggesting that ToV M protein possesses an ER-retention mechanism [196]. Although these were preliminary data, BToV M protein with HA tags at the C-terminus showed similar behaviours in M-expressing COS7 cells, whereas N-terminal tagging resulted in significantly different subcellular localisations [200]. Therefore, the N-terminal ectodomain of BToV M protein may be involved in its intracellular accumulation, unlike the CoV M protein [200].

Most CoV M proteins were indispensable but insufficient for VLP formation, and the co-expression of M and E proteins (minimum viral protein requirement in most CoV VLPs) was required for VLP formation [171,201], the efficiency of which was increased by N protein [87]. By contrast, the minimum viral protein requirement for ToV VLP formation is unknown. Our repeated attempts to produce ToV VLP by co-expression of BToV M/N or M/N/S proteins using a virus-free plasmid-based system in HEK293T cells have been unsuccessful [202]. Although it is unclear whether these failures were as a result of incorrect expression viral protein ratios or the involvement of other viral or cellular proteins and factors, the absence of the E-like protein of ToV predicted the requirement for other proteins to compensate. U1 protein, which might have CoV E protein-like function, may facilitate ToV VLP formation.

### 6.4. Nucleocapsid (N) Protein

**Figure 6:**
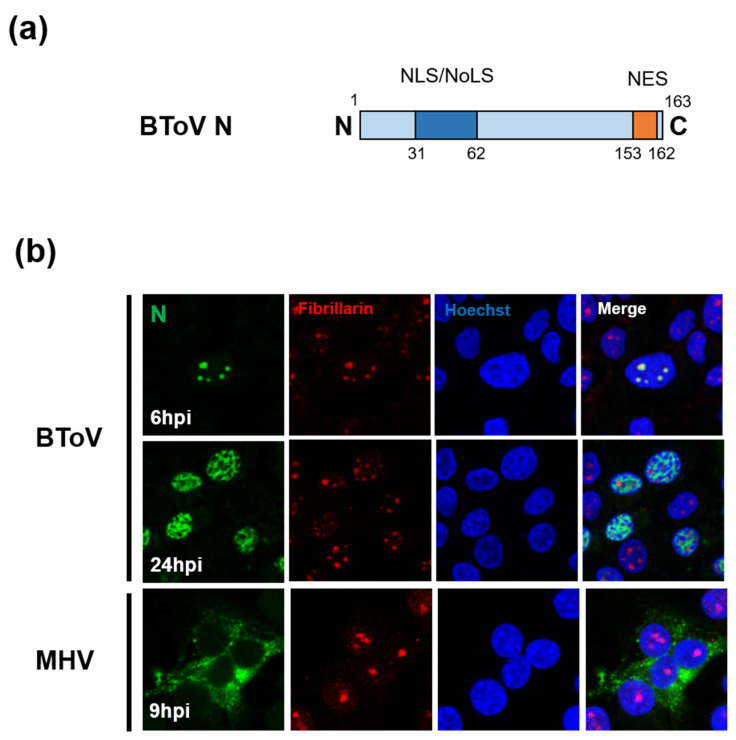
(**a**) Schematic diagrams of the N protein of ToV, with two nuclear/nucleolar localisation signals (NLS/NLoS) and nuclear export signals (NES). (**b**) Subcellular localisation of BToV or MHV (CoV) N proteins in infected cells. The cells were fixed and permeabilised at the indicated hours post-infection (hpi). N proteins and nucleolar marker fibrillarin were detected using mouse anti-N antiserum (green) and anti-fibrillarin rabbit mAb (red), respectively. The nucleus was stained with Hoechst solution (blue).

The N protein of ToV (~20 kDa, ~167 aa) is the most abundant protein in the virion (about 80% of total viral protein mass), is phosphorylated, and has RNA-binding activity [203,204]. The size of ToV N protein is less than half that of CoV N protein (~450 aa). Although the primary function of N protein is to package the viral genome into the nucleocapsid, CoV N protein has been reported to have multiple functions, not only in the viral life cycle, such as replication, transcription and translation, but also in the host cell response such as cell-cycle regulation, INF antagonism, and translational shutoff [205]. Whether ToV N protein has the same functions as CoV N protein is unknown, but an interesting difference between ToV and CoV N proteins is their subcellular localisation (Figure 6b). Because positive-stranded RNA viruses replicate in the cytoplasm, their structural proteins are generally transported there. However, N proteins of CoV and ToV are partially or predominantly localised in the nuclear compartments. CoV N proteins were predominantly localised in the cytoplasm, but in some cases partially in the nucleolus [206,207]. In contrast, studies of EToV N proteins have yielded conflicting results: one reported that they accumulated mainly in the nucleus [66], and the other that they were localised mainly to the cytoplasm [208]. A later study showed that BToV N protein was transported to the nucleolus during early infection, but predominantly in the nucleoplasm during late infection. Remarkably, a small amount of BToV N protein was present in the cytoplasm during infection (Figure 6b) [67]. Conventional EM studies have reportedly detected tubular nucleocapsids, probably formed by N proteins, in both the nucleus and cytoplasm of infected cells [4,5].

The CoV and ToV N proteins have nucleocytoplasmic trafficking signals, including nuclear localisation signals (NLSs) and nuclear export signals (NESs), and poorly defined nucleolar localisation signals (NoLSs). The NESs have several consensus sequences recognised by a chromosomal maintenance 1 (CRM1) protein, and the export of proteins dependent on these NESs is inhibited by leptomycin B (LMB). In CoV, the NLS/NoLS and NES of IBV and porcine epidemic diarrhea virus (PEDV) have been identified [209,210,211]. PEDV N protein contains overlapping NES consensus sequences that were sensitive to LMB [211], whereas IBV N protein possesses another NES consensus sequence, but its export from nuclear compartments was not inhibited by LMB, suggesting that the nuclear export mechanism is CRM1-independent [210]. BToV N protein also contains NLS/NoLS at the N-terminus and NES at the C-terminal end (Figure 6a), and its NES functions in a CRM1-independent manner, despite complete correspondence to the CRM1-dependent NES consensus sequence, like IBV N proteins [67].

The nuclear or nucleolar function(s) of N proteins are unclear, but CoV N protein in the nucleolus may be involved in cell division. IBV N protein, which colocalised with the major nucleolar protein fibrillarin and interacts with nucleolin, delayed cell growth by disrupting cytokinesis [212]. TGEV N protein arrested the S and G2/M phases of the cell cycle, suppressing cell proliferation and apoptosis [213]. By contrast, the N protein of porcine reproductive and respiratory syndrome virus (PRRSV), an arterivirus evolutionarily related to CoV and ToV, was transported to the nucleolus via NLS/NoLS [214] and co-localised and interacted with fibrillarin [215]. Recombinant PRRSV with mutations in its NLS/NoLS, which lost its nucleolar accumulation, was successfully rescued, but slowed growth and decreased pathogenicity [216,217]. In contrast, recombinant BToV with a mutation in the NLS/NoLS of N protein, which lost signal-mediated nuclear accumulation, was not successfully rescued, suggesting that NLS/NoLS of BToV N protein is essential for viral growth [67]. The importance of N-nuclear accumulation in viral growth may be explained by the unique characteristics of ToVs. EToV replication but not that of CoVs and arteriviruses was inhibited by actinomycin D (an inhibitor of cellular DNA transcription) and alpha-amanitin (an inhibitor of DNA-dependent RNA polymerase II), suggesting that ToV replication requires nuclear functions or specific cellular genes [190]. Elucidation of the nucleolar/nuclear functions of ToV N protein should be addressed in a future study. Moreover, despite being the major component of the nucleocapsid, BToV N protein accumulates predominantly in the nuclear compartments during infection. To be incorporated into virions, nucleocapsids must be transported to the vicinity of the budding site, Golgi apparatus (or ER). Therefore, analysis of the cytoplasmic transport and virion-incorporation mechanisms of nucleocapsids formed by ToV N protein will provide insight into different virion assembly mechanisms of ToV and CoV.

## 7. Reverse Genetics

Reverse genetics systems of CoVs and ToVs provide powerful tools for studying their fundamental viral life cycles and pathogenesis, or to support vaccine or anti-viral drug development. However, large genome sizes complicate their manipulation, and the instability of some CoV replicase genes in bacteria have been a serious obstacle to the development of full-length infectious cDNA clones [218]. These obstacles were overcome through the innovation and extraordinary effort of three laboratories that developed methods such as the bacterial artificial chromosome (BAC) [219], in vitro ligation of cDNA fragments [220], and vaccinia vector with full-length CoV cDNA [221]. Recently, transformation-associated recombination cloning into a yeast artificial chromosome was developed as a fourth new method [222]. These technologies have facilitated the establishment of reverse genetics systems for many human and animal CoVs [218,223], including the recently emerged SARS-CoV-2 [224]. These reverse genetics approaches have been used extensively to study the roles of viral proteins in viral replication, pathogenesis, host innate immune systems, cell and tissue tropism, and anti-CoV drug screening and vaccine development [223]. Although ToV reverse genetics was not established until very recently, a reverse genetics system using a full-length infectious cDNA clone of BAC-based BToV has finally been developed. Using this system, several recombinant BToVs with HE or N gene mutations have been successfully recovered and analysed [24,67].

Assembly of BToV cDNA fragments into a BAC to generate a full-length cDNA clone was conducted using the Red/ET recombination method, which is more rapid and efficient than traditional methods [225]. However, this method can result in the insertion of an unintended *E. coli*-derived sequence, which may counter toxic regions in bacteria, into the BToV sequence of the BAC. Similar phenomena were not observed in the full-length genome assembly of PEDV and FCoV into a BAC using the same method [226,227], but were observed in the subcloning of a TGEV cDNA fragment into high-copy plasmids [220]. In both cases, the inserted positions were similar (ca. 10,000 nt), suggesting that these regions (BToV is within a 3CL^pro^) are particularly toxic in bacteria.

Because recombinant viruses carrying reporter genes such as green fluorescent protein and luciferase genes are useful for studying the fundamental viral life cycle and for screening therapeutic compounds, several recombinant CoVs carrying these reporters have been generated [222,226,227,228]. In these recombinant CoVs, accessory genes were replaced with reporter genes, all of which were capable of stable expression of the reporter protein, with similar or slightly lower growth ability than the parent virus. In contrast, recombinant BToV in which the accessory HE gene was replaced with the enhanced green fluorescent protein (EGFP) gene (BToV^EGFP^) could express an EGFP protein, but with significantly reduced growth compared to the parental wild type BToV, and lower EGFP expression. Moreover, the BToV^EGFP^ was found to lose the EGFP gene easily, after only one passage. Interestingly, a BToV^EGFP^ variant with markedly higher EGFP expression and growth ability was identified and isolated during serial passages, although it eventually lost the EGFP gene [24]. Because the isolated BToV^EGFP^ variant contains several nsp mutations, which may contribute to EGFP gene acceptance, these mutations are currently under analysis.

## 8. Concluding Remarks and Future Perspectives 

ToV research has progressed little compared to CoV research because only one EToV (BEV) was successfully propagated in cultured cells for many years, and because ToV infections are usually asymptomatic or non-lethal. However, BToVs have been detected worldwide, causing economic losses due to calf diarrhoea. PToV has also spread globally, and has not caused great economic losses, but its symptoms can increase in severity due to co-infections. In addition, frequent inter- or intra-recombination can cause increased pathogenesis or unpredicted host adaptation. These factors have raised awareness of ToVs as important pathogens and highlight the need for further basic research on ToVs. Therefore, to promote further research and understanding of ToVs and to contribute, ultimately, to their control, we reviewed the recent progress in ToVs, which occur worldwide but are under-investigated, as notable enteric viruses. The following summarises what we have learned or achieved in the last 10~20 years.

- In addition to BToV, PToV is widespread globally, while the occurrence of HToV is not clear.
- An ultrastructural study examined the membranous structures in ToV-infected cells. ToVs induce DMVs, but not additional membrane structures, such as the CMs, zippered ER, or DMSs observed in CoV.
- There is frequent inter- and intra-recombination in BToV and PToV. Moreover, an EV-G that obtained ToV-like PL^pro^ via heterologous recombination has been detected in many countries.
- ToVs use a transcription strategy unique among the nidoviruses, and use combined discontinuous/continuous transcription to synthesise a set of sg mRNAs.
- Two deduced ORFs encoding proteins of unknown function, U1 and U2, translated from an unconventional CUG initiation codon, are found in the 5’ -UTR and within ORF1a in the genome.
- The N proteins of BToV, which replicates in the cytoplasm, predominantly accumulate in the nuclear compartments during all infection processes, despite being a main structural protein. The different subcellular localisation of N proteins suggests a different virion assembly mechanism in ToV and CoV.
- The three-dimensional structures of BToV and PToV HE proteins have been resolved, and their substrate specificities characterised.
- BToV has been isolated and propagated in HRT18 cells.
- A reverse genetics system for BToV has been established.

The successful isolation of several BToVs in cultured cells and the establishment of a BToV reverse genetics system will be the driving force for future ToV research. In particular, reverse genetics will be applied to address the following open issues or support detailed studies.

- Research to be conducted on the transcriptional mechanism of ToVs includes the following:
  ➢ To determine whether the highly conserved sequence (CUUUAGA) of L-TRS and each B-TRS is actually intolerant of mutations, as suggested by initial observations.
  ➢ To determine whether complementarity between L-TRS and anti-B-TRS is required for ToV replication, since the template switch driven by base pairing between L-TRS and anti-B-TRS does not occur in ToV.
  ➢ To determine whether the highly conserved L-TRS and B-TRS have other roles in this region beyond acting as a terminator signal on genome (i.e., extended TRS:CACN_3–4_CUUUAGA) and promoter signal of sg mRNA (ACN_3–4_CUUUAGA).
  ➢ To determine what the structure and sequence of HP, and the 23-nt homology sequence following L-TRS and HP play roles in the discontinuous transcription of mRNA2.
  ➢ To determine whether discontinuous transcription is inhibited by introducing B-TRS upstream of the S gene or insertion of non-coding intergenic regions including B-TRS between the ORF1b and S genes, and, if so, to analyse the phenotype of the recombinant ToVs.
- Further study should determine whether U1 and U2 proteins are actually translated from unconventional CUG initial codons in infected cells. If so, their functions in virus infection and the protein(s) essential for viral growth in infected cells should be identified.
- The roles of these functional domains in nsps conserved in nidovirals have been extensively studied using CoVs. However, whether the knockout recombinant BToVs of the corresponding domains of ToVs show the same phenotype remains unknown.
- The ligand and substrate specificity of ToV HE are 9-O-Ac-Sias; however, the substrate specificity of the ToV S protein in Sias-mediated hemagglutination activity remains unknown. Further study should also be conducted to determine whether the ToV S and HE proteins are functionally related.
- The function of the region of structural proteins that significantly differs from that of CoVs, such as the CT of the S protein, should be identified, along with the phenotype of a recombinant virus with mutations in this region.
- BToV N proteins accumulate predominantly in nuclear compartments during infection. The nuclear or nucleolar function of BToV N proteins should be investigated.
- The ease and efficiency of analysing cell entry mechanisms or anti-viral drug screening of ToVs should be improved using recombinant ToVs carrying reporter genes.
- Reverse genetics can provide attractive new ideas and strategies for the development of new vaccines. For example, a recombinant BToV in which the HE gene is replaced with the S1 region of BCoV containing the major antigenic determinants may become a bivalent vaccine that protects from both BToV and BCoVs.

Furthermore, the following challenging research topics remain:

- Identification of the functional receptors of ToVs.
- Resolution of the three-dimensional structure of major proteins such as the S protein.
- Analysis of the full-genome sequence and successful cell isolation of HToV, which will definitively prove its existence.

Due to the close evolutionary relationship between ToVs and CoVs, ToV research is expected to bridge the missing link in the evolution of nidovirus. Nevertheless, studies of the transcriptional mechanisms, and 3CL^pro^ and N protein subcellular localisation of ToV have highlighted differences from CoVs and other nidovirus, further complicating the sequence of nidoviral evolution. Future studies of ToVs will provide interesting new insights into the fundamental viral processes of this neglected pathogen, as well as nidoviral evolution.

## Data Availability

Not applicable.
